# Hedgehog Pathway as a Potential Intervention Target in Esophageal Cancer

**DOI:** 10.3390/cancers11060821

**Published:** 2019-06-13

**Authors:** Da Wang, Peter W. Nagle, Helena H. Wang, Justin K. Smit, Hette Faber, Mirjam Baanstra, Arend Karrenbeld, Roland K. Chiu, John Th.M. Plukker, Robert P. Coppes

**Affiliations:** 1 Department of Biomedical Sciences of Cells and Systems, Section Molecular Cell Biology, University of Groningen, University Medical Center Groningen, 9700 RB Groningen, The Netherlands; daisywangda@hotmail.com (D.W.); p.w.k.nagle@umcg.nl (P.W.N.); h.h.wang@umcg.nl (H.H.W.); m.baanstra@umcg.nl (M.B.); r.k.chiu@rug.nl (R.K.C.); 2 Department of Radiation Oncology, University of Groningen, University Medical Center Groningen, 9700 RB Groningen, The Netherlands; 3 Department of Surgery, University of Groningen, University Medical Center Groningen, 9700 RB Groningen, The Netherlands; j.k.smit@umcg.nl (J.K.S.); j.t.m.plukker@umcg.nl (J.T.M.P.); 4 Department of Pathology, University of Groningen, University Medical Center Groningen, 9700 RB Groningen, The Netherlands; a.karrenbeld@umcg.nl

**Keywords:** Esophageal cancer, Hedgehog pathway, cancer stem cells, treatment resistance

## Abstract

Esophageal cancer (EC) is an aggressive disease with a poor prognosis. Treatment resistance is a major challenge in successful anti-cancer therapy. Pathological complete response after neoadjuvant chemoradiation (nCRT) is low, thus requiring therapy optimization. The Hedgehog (HH) pathway has been implicated in therapy resistance, as well as in cancer stemness. This article focusses on the HH pathway as a putative target in the treatment of EC. Immunohistochemistry on HH members was applied to EC patient material followed by modulation of 3D-EC cell cultures, fluorescence-activated cell sorting (FACS), and gene expression analysis after HH pathway modulation. Sonic Hedgehog (SHH) and its receptor Patched1 (PTCH1) were significantly enriched in EC resection material of patients with microresidual disease (mRD) after receiving nCRT, compared to the control group. Stimulation with SHH resulted in an up-regulation of cancer stemness in EC sphere cultures, as indicated by increased sphere formation after sorting for CD44+/CD24− EC cancer stem-like cell (CSC) population. On the contrary, inhibiting this pathway with vismodegib led to a decrease in cancer stemness and both radiation and carboplatin resistance. Our results strengthen the role of the HH pathway in chemoradiotherapy resistance. These findings suggest that targeting the HH pathway could be an attractive approach to control CSCs.

## 1. Introduction

Despite modern advances in the treatment of esophageal cancer (EC), most patients still face a poor prognosis primarily due to locoregional advanced disease, metastases at the time of diagnosis and early recurrences after initial curative treatment [1]. Esophageal adenocarcinoma (EAC) arising from gland tissue is one of the two main histological EC types and has the fastest growing incidence in the western world, increasing 300% within the last 30 years [2]. EAC is currently the predominant type in the western world beyond esophageal squamous cell carcinoma (ESCC), which arises from the epithelium [2,3]. If resectable, both types are commonly treated with neoadjuvant chemoradiotherapy (nCRT) followed by surgery. However, only 29% of these tumors will reach a pathologic complete response (pCR), while >50% respond partially and around 20% will not respond at all to nCRT [4]. Moreover, most patients will relapse within 2.5 years [5]. These numbers indicate that there is an urgent need for treatment optimization. Elucidating tumor regulatory mechanisms that are on the basis of sustained disease might provide new therapeutic targets. Furthermore, it may provide tools for developing novel ways to predict therapy response preventing patients from undergoing unnecessary therapy.

The Hedgehog (HH) pathway, which plays a major key role in embryogenesis, is essential for tissue development and differentiation [6,7]. In adult tissue, the HH pathway is inactive in normal tissues except for stem and progenitor cells where it plays a role in the maintenance of homeostasis and tissue repair [6,7]. Three mammalian HH ligands have been described, namely Sonic Hedgehog (SHH), Desert Hedgehog (DHH) and Indian Hedgehog (IHH). Of these three ligands, SHH is by far the best characterized [6,7]. In the presence of SHH ligand, SHH binds to the Patched1 (PTCH1) transmembrane receptor relieving the inhibitory effect on Smoothened (SMO). Consequently, a cascade of reactions leads to the activation of GLI family zinc finger 1,2 and 3 transcription factors (GLI1, GLI2 and GLI3). Upon binding of the GLI transcription factors to a protein complex including Suppressor of Fused (SUFU) (an essential component of HH signaling in vertebrates [8]), GLI transcription factors translocate to the nucleus where they control the transcription of target genes [7,9,10]. PTCH1 is transcriptionally induced upon activation of SMO along with the other pathway targets. Target genes, such as PTCH1 and GLI1, play a role in maintaining the regulation of the pathway itself, while others promote cell survival. Aberrant activation or mutation in the key members of the HH pathway is associated with the development of cancer and resistance to conventional anticancer therapy [7,9]. Moreover, the HH pathway has been linked to a population of cells called cancer stem-like cells (CSCs) which is a major contributor of therapy resistance [11,12,13], while PTCH1 is often associated with cancer stemness [14]. It has previously been shown that CSCs possess features of normal stem cells such as the ability to self-renew and to generate all other cells of the tumor [11,12,13], and may play an important role in tumor-initiation, metastases, recurrences and therapy resistance in different kinds of cancers [15]. Therefore, this population of cells is known for its aggressiveness and should be eradicated. Previously, Smit et. al. [16] showed that a subpopulation of a commonly used CSC marker CD44 expressing cells the CD44+/CD24− expressing cells, displayed more potently CSC characteristics. In vitro, CD44+/CD24− cells yield significantly more spheres and are more resistant to irradiation. In vivo, this population forms tumors earlier which also grow larger in size. Serial transplantation experiments of first, second and third generation tumors demonstrated a strong correlation between the proportion of CD44+/CD24− cells and the in vivo growth rate [16]. Furthermore, CD44+/CD24− was found to be present in 50% of pretreatment tumor biopsies of patients with residual EAC after treatment in surgical specimens but absent in all biopsies of patients who had a pathological complete response after nCRT [16]. These results suggest that CSCs are more enriched in the CD44+/CD24− population compared to CD44+/CD24+ and the unsorted population, and could be used as a readout for esophageal CSCs. Interestingly, the combination CD44+/CD24− is also commonly used to isolate breast CSCs [17].

Thus, studying the HH pathway in EC may reveal a new way to eradicate CSCs leading to reduced therapy resistant tumors. Our aim is to explore whether the HH pathway can be considered as a potential target for future anti-CSC EC therapy.

## 2. Results

**Figure 1:**
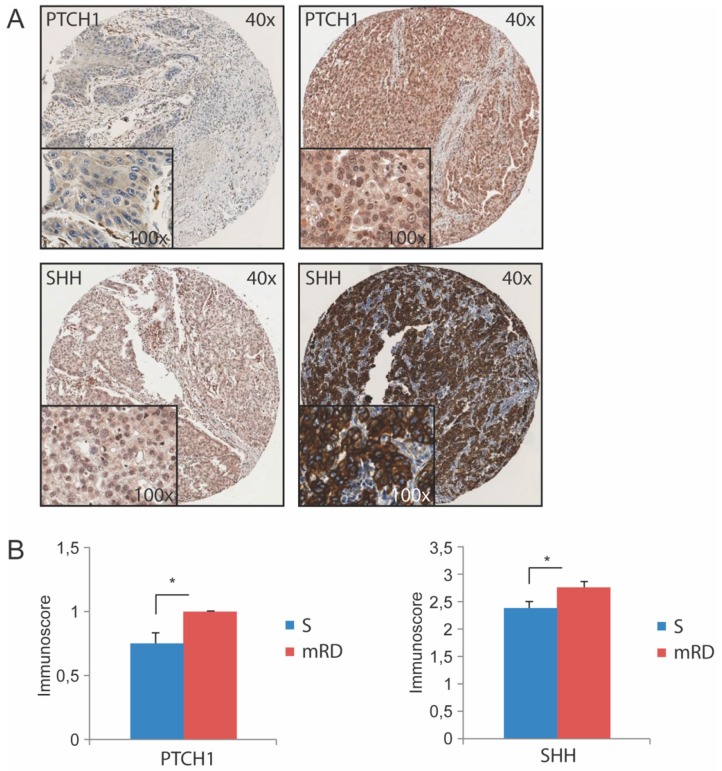
PTCH1 and SHH are up-regulated in mRD patients compared to surgery alone patients (S). (**A**) Representative samples of low intensity PTCH1 and weakly positive SHH expression (respectively upper left and lower left), and high intensity PTCH1 and strong positive SHH expression (respectively upper right and lower right). (**B**) Comparison of PTCH1 (*p* = 0.04) and SHH (*p* = 0.04) IHC expression between mRD after neoadjuvant CRT resection specimens (*N* = 16) and S specimens (*N* = 32). Error bars represent standard error of the mean (SEM), *p < 0.05.

To investigate if the HH pathway is involved in regulating EC CSCs, the level of expression of the HH members PTCH1 and SHH were assessed in EC resection material from patients that underwent a surgical resection in our center during the period 2006 and 2011. Sixteen tumor tissue specimens from non or marginally responding EC patients to nCRT (microresidual disease, mRD group with Mandard tumor regression grade 4 and 5) were compared to 32 matched control tumor specimens of patients who only had undergone radical surgery (S group). The latter group with unknown therapy responsiveness represents a mix of all potentially responding groups. Indeed, both PTCH1 and SHH were significantly higher expressed in mRD tissue compared to S tissue (both *p* = 0.04, Figure 1A,B). These results indicate that the HH pathway may be related to therapy resistance. 

**Figure 2:**
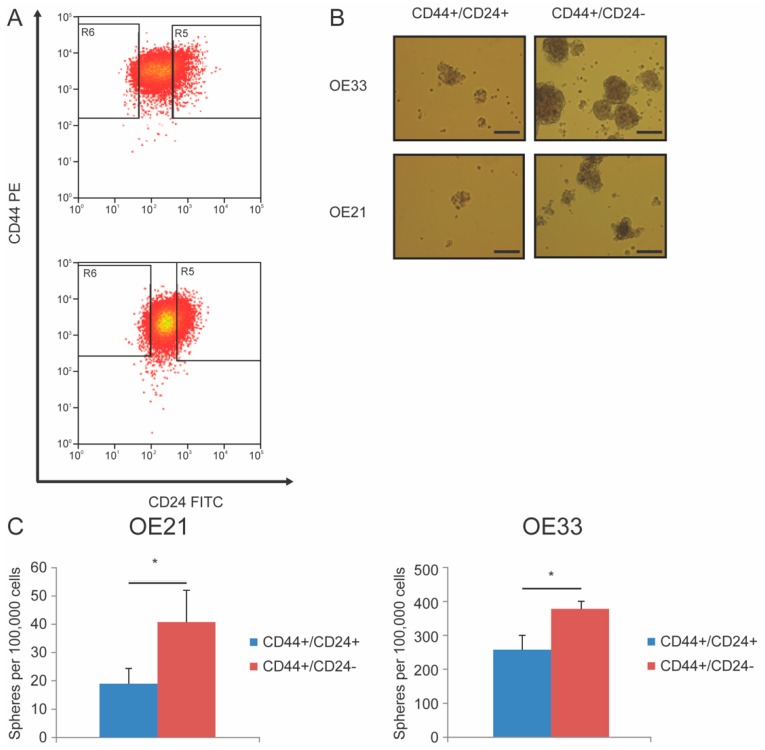
Enhancement of sphere formation capacity by sorting for CD44+/CD24−. (**A**) Representative FACS plots of OE21 and OE33 stained with CD24 FITC and CD44 PE. (**B**) Representative images of spheres. Bar indicates 100 µm. (**C**) Quantification of spheres shown in (**B**). OE21 CD44+/CD24− vs. CD44+/CD24+ (*p* = 0.01) and OE33 CD44+/CD24− vs. CD44+/CD24+ (*p* = 0.02, *N* = 3) spheres after five days of culture. Error bars represent standard deviation, *p < 0.05.

To validate the impact of the HH pathway in the regulation of CSCs that might play a fundamental role in the observed therapy resistance in patient derived tumor resection material, CD44+/CD24− was taken as a read-out for cancer stemness after first verifying the sphere forming ability, as a hallmark of CSCs, of this population in vitro. The sphere forming potential of two subpopulations of cells were isolated by sorting the 3–15% of the outer extreme of CD44+/CD24− CSC population and CD44+/CD24+ non-CSC population, in OE21 and OE33 EC cell lines isolated at 70% confluency (Figure 2A). Five percent of the outer extreme of each subpopulation were isolated by FACS. Indeed, as shown previously [16], the CD44+/CD24− population formed significantly more spheres when compared to CD44+/CD24+ population in both OE21 and OE33 cell lines (*p* = 0.01 and *p* = 0.02 respectively, Figure 2B,C), which is also known to be resistant to radiation and form tumors more potently as shown previously [16].

**Figure 3:**
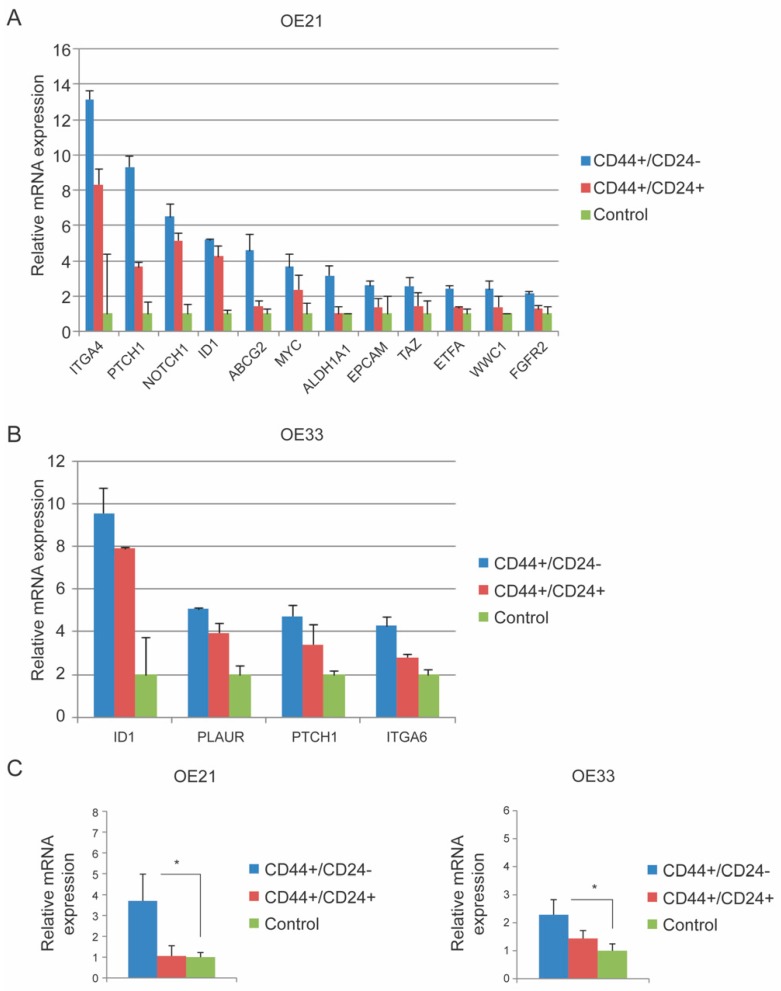
PTCH1 is up-regulated in CD44+/CD24− CSC population in both OE21 and OE33 cell lines according to a qPCR array of 84 genes related to cancer stemness. (**A**) Relative mRNA expression of CSC related genes in OE21 CD44+/CD24− CSC population, CD44+/CD24+ population and control (obtained from more differentiation form the cell lines derived xenograft tumors, [16]). Shown genes express >2 fold in CD44+/CD24− population compared to control. Control is set on 1. (**B**) Relative mRNA expression of CSC related genes in OE33 CD44+/CD24− CSC population, CD24+/CD44+ population and control. Shown genes express >2 fold in CD44+/CD24− population compared to control. (**C**) Validation of relative mRNA expression of PTCH1 in CD44+/CD24− CSC population, CD24+/CD44+ population and control in OE21 and OE33 by qPCR. PTCH1 was 3.8 fold up-regulated in CD44+/CD24− CSC population compared to control in OE21 (*p* = 0.04, *N* = 3) and 2.3 fold in OE33 (*p* = 0.04, *N* = 5). Error bars represent standard deviation.

Next, in order to identify a pathway in an unbiased way that may be involved in the regulation of the CD44+/CD24− CSC population, a qPCR array of 84 genes related to cancer stemness was performed on the subpopulations of both OE21 and OE33 cells and were compared to previously harvested OE21- and OE33-derived xenograft tumor controls representing a more differentiated population [16]. In this way, genes that were up-regulated in CD44+/CD24− expression compared to the control could be identified (Supplementary Table S1 and Figure 3A). Genes that had >2-fold upregulation compared to controls were considered to be upregulated. In OE21, multiple genes were >2-fold upregulated including PTCH1, a member of the HH pathway. In OE33, fewer genes displayed >2-fold upregulation, among which was found to be again PTCH1 (Supplementary Table S1 and Figure 3B). To narrow down genes, the differences of expression between the CD44+/CD24− population and CD44+/CD24+ population were compared to their controls. Interestingly, PTCH1 was upregulated in CD44+/CD24− and CD44+/CD24+ populations in both cell lines compared to their controls (OE21 9.31 and 3.67, and OE33 2.37 and 1.71 respectively). These findings were validated by separate qPCR analyses (both cell lines *p* = 0.04, Figure 3C).

**Figure 4:**
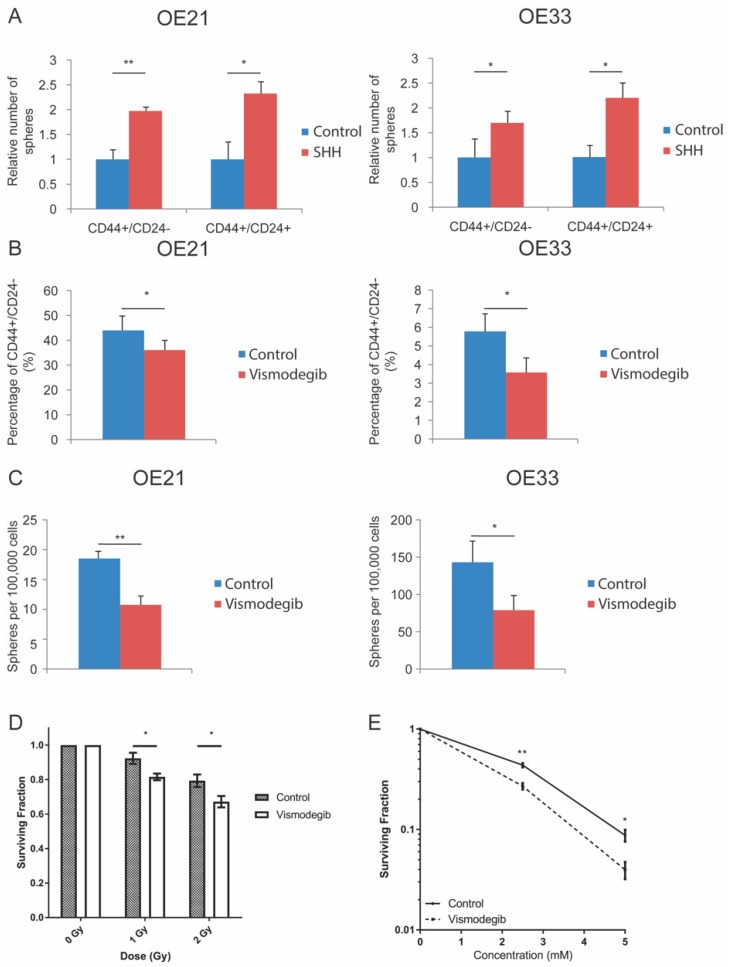
Sonic Hedgehog, ligand of the PTCH1 receptor up-regulates sphere formation of both CD44+/CD24− and CD44+/CD24+ populations in OE21 and OE33 while vismodegib, a HH pathway inhibitor, deselects for the CD44+/CD24− population, decreases sphere formation and increases radiosensitivity. (**A**) Relative number of spheres formed in OE21 and OE33 after sorting for CD44+/CD24− and CD44+/CD24+ populations with the controls set on 1. Cells treated with SHH formed significantly more spheres in CD44+/CD24− and CD44+/CD24+ populations compared to their controls (DMSO-treated) (OE21: 1.97 (*p* = 0.009, *N* = 3) and 2.33 (*p* = 0.03, *N* = 3) fold respectively. OE33: 1.68 (*p* = 0.04, *N* = 3) and 2.18 (*p* = 0.04, *N* = 3) fold respectively. Spheres were counted after five days. (**B**) Percentage of CD44+/CD24− expression in OE21 and OE33 (unsorted populations) control (DMSO-treated) and vismodegib (5 nM) treated cells. Vismodegib treated cells showed significantly a deselection of the CD44+/CD24− phenotype in both cell lines compared to (DMSO-treated) controls (OE21 *p* = 0.02, OE33 *p* = 0.04, *N* = 4). (**C**) Amount of spheres formed in OE21 and OE33 control and vismodegib (5 nM) treated cells. Spheres were significantly lower in vismodegib treated cells compared to control (DMSO-treated) in OE21 (*p* = 0.008, *N* = 3) and OE33 (*p* = 0.03, *N* = 4). Survival of vismodegib-treated cells in response to (**D**) radiation and (**E**) Carboplatin. Error bars represent standard deviations, *p < 0.05, **p < 0.01.

To assess the role of the HH pathway in CSC regulation, the HH pathway was modulated by exogenously adding its ligand SHH (0.4 µg/mL) to CD44+/CD24− and CD44+/CD24+ cells after sorting (similar gating strategies as shown in Figure 1A were used, Supplementary Figure S1A) in both OE21 and OE33. After adding, SHH induced a significant increase in sphere forming potential in both CD44+/CD24− and CD44+/CD24+ sorted cells when compared to control in OE21 and OE33 (Figure 4A), whereas the solvent (PBS + 0.1% BSA) did not affect the sphere forming ability of either cell line (data not shown). In contrast, inhibition of the HH pathway using vismodegib (5 nM; a concentration chosen based on the viability of cells treated with vismodegib alone (Supplementary Figure S2)) resulted in a reduction of the CD44+/CD24− CSC phenotype in OE21 and OE33 cells when compared to controls (Figure 4B, FACS gating strategies are shown in supplementary Figure S1B). Moreover, sphere forming ability also decreased after vismodegib treatment in both OE21 and OE33 cells (Figure 4C). Compared to other cells within a tumor, it has previously been shown that CSCs are more resistant to many forms of cancer treatments [15]. Therefore, modulation of the CSC population should illicit a change in radioresistance in OE-21 cells [16]. To this end, OE-21 cells were photon irradiated or treated with carboplatin (a common chemotherapeutic drug used for esophageal cancer treatment) following 48 hours treatment with vismodegib and their clonogenic capability was assessed. Indeed, vismodegib-treated cells were found to be more sensitive to both radiation and carboplatin treatment than control cells (Figure 4D,E). These findings indicate that the HH pathway may be involved in the regulation of the CD44+/CD24− CSC population increasing cancer stemness and as a consequence may result in an increased therapy resistance. Inhibiting the pathway with vismodegib may be of therapeutic value and should be explored further.

## 3. Discussion

In this study, we demonstrated that the HH pathway is up-regulated in nCRT patients with Mandard 4 and 5 statuses after reviewing the resection material and moreover it may be a key player in the regulation of cancer stemness as shown by the change in the amount CD44+/CD24− CSC population upon HH pathway modulation. The first observation can be explained by the fact that nCRT may activate the HH pathway and in turn making cells acquiring more CSC features including the therapy resistance characteristic [18]. Another clarification for this phenomenon could be that a subset of cancer cells already has an activated HH pathway prior to therapy that renders them to survive chemo- and radiotherapy [19,20,21,22]. Most likely, a combination of both may occur. The S group contains patients from <2006 when radical surgery was the standard treatment for esophageal cancer implicating that this group includes non-responders, partial responders and complete responders. This could be the reason why the results of PTCH1 and SHH expression between this group and the mRD group are more condensed. Next, we verified these results in an unbiased qPCR array experiment with sorted CD44+/CD24− OE21 and OE33 cells compared to sorted CD44+/CD24+ OE21 and OE33 cells, and dissociated OE21 and OE33 cells previously harvested from mouse xenograft tumors [16]. Cells from xenografts represent a more differentiated and heterogeneous group of cells, and thus represent all four populations of CD44 and CD24 following FACS analysis. Therefore, the xenograft group was considered as our control group, as the original cell lines did not show expression of the CD44−/CD24+ or CD44−/CD24−. On the other hand, cells obtained from xenografts may be influenced by tumor microenvironmental factors, such as hypoxia activating, a number of CSC-related pathways including the HH pathway. However, our qPCR data show that PTCH1 expression is lower in our control group compared to the other two groups indicating that even with the potential influence of the tumor microenvironment, PTCH1 expression is less compared to a ‘pure’ CD44+/CD24− population suggesting the tumor is indeed composed of more differentiated cells. Co-labelling CD44, CD24 and PTCH1 for flow cytometry purposes was not favorable due to the lack of conjugated antibodies against PTCH1. While vismodegib binds directly to PTCH and SMO and thereby inhibits the activation of GLI [23], the effect of vismodegib treatment on the expression of other genes which were found to be upregulated in the CSC subpopulation but not directly involved in the HH pathway (Figure 3) was not investigated. It would be interesting to determine if the upregulation of these genes was also abrogated upon vismodegib treatment, as this may indeed further confirm that the CSCs in general have been targeted. RNA-seq analysis may reveal other interesting targets not present on our cancer stemness dedicated array.

To date, we are the first to show the importance of the HH pathway in controlling cells in a specific CSC population in EC. The activation of the HH pathway in subsets of EC has been shown previously [19,20,21,22] and is in line with our study. In addition, some studies have shown the association of the HH pathway with therapy resistance but did not address the involvement of specific CSC populations [20,22]. We have demonstrated that inhibiting the HH pathway is related to a reduction of cells with the cancer stem cell related phenotype (CD44+/CD24−), less sphere forming capability and more radiosensitivity. Although vismodegib is being tested in the clinic, xenograft models in combination with HH pathway modulation will provide more information on the potential therapeutic effectiveness. Furthermore, it would be important for future studies to confirm if the effect was indeed directly due to the actions of vismodegib on the HH pathway using other HH-targeting molecules which are currently being used in clinical trials, such as LDE225 [24,25,26].

Vismodegib, also known as GDC-0449, is a small molecule HH inhibitor that blocks the interaction between the PTCH-receptors and their ligands [27]. The choice of vismodegib is based on its advantageous characteristics. It is more potent than the better known cyclopamine and has more favorable pharmaceutical characteristics. Moreover, it has already been FDA approved for advanced basal cell carcinoma [27]. Vismodegib is currently used in early trials for treatment of basal cell carcinomas and medulloblastoma, showing promising early indications in enhancing treatment responses [28,29,30]. Therefore, vismodegib could be a promising drug to target esophageal CSC populations.

## 4. Material and Methods

### 4.1. Patient Material

Sixteen tumor tissue specimens of microscopic residual disease (mRD group) from EC patients who showed little or no response to nCRT (Mandard tumor regression grade 4 and 5) following surgical resection at the University Medical Center Groningen, during the period 2006 and 2011 were included in the primary study group. Only patients with histologically proven ESCC or EAC after curative resections were included. Some patient samples were excluded due to lack of sufficient tissue. Furthermore, all patients had to be treated with nCRT according to the CROSS regimen [4,5]. These 16 tumor specimens were matched (1:2) to 32 control tumor specimens of patients who have only undergone surgery (S group). The S group contains patients from the period before 2006 when radical surgery alone was considered as standard therapy for EC. When extrapolating this group, it will represent the whole treatment group of current nCRT, including none, partial and complete responders. Matching was based on histology (EAC or ESCC) and depth of tumor invasion (T-stage). Tissue microarrays (TMAs) were constructed as previously described [16]. The study was conducted according to the guidelines of our Ethical Institutional board (www.ccmo.nl). Archival tissue was handled according to the Dutch Code for proper use of Human Tissue (www.federa.org).

This study did not require an ethics approval or a consent to participate. The human material used in this study was archival tissue. The study was conducted according to the guidelines of our Ethical Institutional board (www.ccmo.nl). Archival tissue was handled according to the Dutch Code for proper use of Human Tissue (www.federa.org). This study was performed in accordance with the Declaration of Helsinki.

### 4.2. Immunohistochemical Staining and Evaluation

Immunohistochemical staining was performed on 5 µm tissue sections from archival material using primary antibodies against PTCH1 (Anti-Patched/PTCH1 antibody Abcam 53715 1:100), SHH (anti SHH 1:100 Abcam 53281) (Abcam, Cambridge, MA, USA), CD44 (anti-mouse/human CD44 Antibody Biolegend Cat.103002 1:100) (Biolegend, London, United Kingdom) and CD24 (anti CD24 Abcam 31622 1:100). The tissue sections were de-paraffinized and subsequently immersed in PBS 2% hydrogen peroxidase to block endogenous peroxidase activity. Antigen-retrieval was performed and the sections were incubated overnight at 4 °C with the primary antibodies. Tissue sections were then incubated with biotinylated secondary antibodies at 1:300 dilutions. The ABC complex was formed using the Vectastain Elite ABC HRP kit (Vector Laboratories, Peterborough, United Kingdom). This complex was visualized with SIGMA FAST 3,3′-diaminobenzidine tablets (Merck KGaA, Darmstadt, Germany). In the final step, sections were counterstained with haematoxylin. Immunohistochemical staining was digitized using Aperio (ASSA ABLOY, Raamsdonksveer, The Netherlands) or Hamamatsu (Almere, The Netherlands). These systems are able to process immunohistochemical slides with high quality, speed and reliability for whole slide imaging. These slides were scored by two independent blinded researchers (D.W. and J.K.S.) using the IRS method without prior knowledge of the clinical outcome. Random samples of each marker were validated by the pathologist A.K. The percentage of positive cells was scored into four categories: (0) no staining, (1) <10%, (2) 10–50%, (3) 51–80% and (4) 81–100%. Intensity was scored as (0) negative, (1) weak, (2) medium and (3) strong. An Immuno-Reactivity Score (IRS) was calculated by multiplying the percentage of positive cells with the intensity score resulting in a score on a scale of 0–12. For evaluation of SHH the IRS was divided into four groups: negative (IRS 0–1, immunoscore 0); weak positive staining (IRS 2–3, immunoscore 1); moderate positive staining (IRS 4–8, immunoscore 2) and strong positive staining (IRS 9–12, immunoscore 3) [31]. Since almost all specimens showed PTCH1 expression in 81–100% of the tumor cells, only intensity for PTCH1 was determined and categorized into negative or low (intensity 0–1, immunoscore 0) and high (intensity 2–3, immunoscore 1).

### 4.3. Cell Lines and Cell Culture

Two esophageal cancer cell lines were used, OE21 derived from ESCC of the upper esophagus and OE33 derived from a poorly differentiated Barrett’s associated tumor of the distal esophagus. Both cell lines were donated by Dr. F.A. Kruyt, Department of Medical Oncology, University Medical Center Groningen and were independently DNA verified as EC cell lines by the Leibniz Institute DSMZ-German Collection of Microorganisms and Cell Cultures (Braunschweig, Germany) in 2012. These cell lines are commonly used in esophageal cancer research. OE21 and OE33 were cultured in GIBCO RPMI 1640 medium (Life Technologies) supplemented with 10% FCS and 1% of penicillin/streptomycin in an incubator of 5% CO_2_ and 37 °C. Both cell lines were passaged in 1:5 at 70% confluency.

### 4.4. Fluorescence Activated Cell Sorting (FACS)

CD44+/CD24− cells and CD44+/CD24+ cells were sorted with both OE21 and OE33 using antibodies directed against CD44 (BD Biosciences, 550989, San Jose, CA, USA) and CD24 (BD Biosciences, 555427) with MoFlo Asterios cell sorter (Beckman Coulter, Woerden, The Netherlands). Isotype controls were used to compensate for aspecific binding of the antibodies. CD24 antibody conjugated with FITC and CD44 antibody conjugated with PE along with their corresponding isotype controls (BD Biosciences, 555748, 555749, respectively) were used. The most left and right (3–15%) population of CD44+/CD24−, respectively, CD44+/CD24+ populations were sorted. 

### 4.5. Sphere Culture and Sphere Count

100,000 cells of each sorted population, CD44+/CD24− and CD44+/CD24+, were seeded in triplicate in each well of a six-well plate with 2 mL of mammocult serum-free medium with supplement (Stemcell Technologies, Vancouver, Canada). Mammocult serum-free medium was used instead of Neural Basal A medium supplemented with N2, bFGF and FGF-2 as previously described by Smit et al. [16]. because of its more potent sphere forming ability in both cell lines without distortion of the results. The wells were coated with agarose to prevent the attachment of cells onto the bottom of the plate. The concentration agarose was 0.023 g/mL dissolved in distilled water. A total volume of 0.2 mL of mammocult was added each day to nourish the cells. To ensure balance of the total amount of formed spheres in both cell lines, 2 × 10^4^ sorted OE33 cells and 1 × 10^5^ OE21 cells were seeded for the modulation of the HH pathway experiments. Pictures of spheres were taken under the microscope after five days. For the quantification, only sphere-like structure in a homogenized aliquot of 100 µL were counted.

### 4.6. HH Pathway Modulation

OE21 and OE33 cells were sorted for CD44+/CD24− and CD44+/CD24+ populations and seeded in each well of a six-well plate coated with agarose to prevent the attachment of the cells onto the bottom of the plate. For OE21 1 × 10^5^ cells were used for each population while 2 × 10^4^ cells were used for OE33 to achieve similar amount of spheres in both cell lines. Each sorted population of both cell lines were cultured in Mammocult medium supplemented by 0.4 μg/mL human recombinant protein Sonic Hedgehog (R and D systems) reconstituted in PBS + 0.1% BSA, according to manufacturer datasheet, to generate spheres. This treated population was compared to its control, which consisted of only Mammocult and PBS + 0.1% BSA. The relative amount of spheres compared to control was calculated after five days. Five nanomoles of vismodegib reconstituted in DMSO was used according to manufacturer instructions (IC50 of 3 nM) and was also based on the biological effect in both cell lines according to viability assays (data not shown). vismodegib also blocks ABC transporter, ABCG2, however only when applied in relatively higher concentrations (IC50 of 1.4 µM) according to the manufacturer datasheet. FACS analyses on LSRII (BD Biosciences) were performed on the expression of CD44+/CD24− and CD44+/CD24+ in OE21 and OE33 after 48 h 5 nM vismodegib treatment in RPMI media and their controls, which consisted of RPMI supplemented with DMSO. Flow cytometry data were analyzed using Kaluza (Beckman Coulter, Woerden, The Netherlands). This software offers flow cytometry analysis software solution designed for high content data. The same cells of both cells lines used in the FACS experiment were seeded in Mammocult medium to generate spheres. This was done immediately after collecting the cells for the FACS analyses to ensure reliability and consistency. For OE21 1 × 10^5^ cells were seeded and for OE33 2 × 10^4^ cells were seeded. Spheres were counted after five days.

### 4.7. RNA Extraction, First Strand Analysis and qPCR

Total RNA was extracted from the sorted cell populations using RNAeasy micro extraction kit (Qiagen, Venlo, The Netherlands). RNA concentration was measured by Nanodrop (Isogen Life Sciences, De Meern, The Netherlands. To ensure RNA quality, standards according to the manufacturer’s datasheet were followed. Subsequently, the first cDNA strand was produced using 500 ng/µL RNA and SuperScript^®^ VILO™ cDNA synthesis kit (Life Technologies, Bleiswijk, The Netherlands). A qPCR array (Qiagen) of 84 genes related to cancer stemness was performed on the CD44+/CD24− and CD44+/CD24+ sorted cells of OE21 and OE33. Additionally, solid tumors generated from the same cell lines obtained from xenografts, as reported previously [16], were used as controls as these populations represent a differentiated group of cells. The xenografts were generated by isolating either sorted CD44+/CD24−, CD44+/CD24+ or unsorted cells OE21 and OE33 cells before injecting either of these populations into animals. These cells were not treated with CRT. These arrays were performed on Biorad CFX connect 96 wells plate according the protocol of the manufacturer. Genes that were >2-fold up-regulated in the CD44+/CD24− CSC population compared to controls were considered relevant. The gene that appeared to be up-regulated in both cell lines was validated by separate qPCR analyses. YWHAZ was used as an endogenous control for normalization. qPCR was performed using SYBR green (Biorad, Lunteren, The Netherlands) to measure the expression of the genes.

### 4.8. Clonogenic Assay

To assess the sensitivity of vismodegib treated cells, a clonogenic assay was performed. Cells were seeded in a 12-well plate at 1 × 10^5^ cells per well and incubated with/without vismodegib (5 nM). After 48 hours, cells were photon irradiated using a 137Cs source (IBL 637 Cesium-137 γ-ray machine). After irradiation cells were washed and harvested from the plate following incubation with trypsin-EDTA. The cells were then counted and seeded in 60 mm cell culture dishes at concentrations ranging from 200–1000 cells depending on the radiation dose or carboplatin concentration. To assess the response to carboplatin following vismodegib treatment, cells were seeded in triplicate at 500 cells per 60 mm cell culture plate at all carboplatin concentrations. For the irradiation experiments, cells were seeded in triplicate at multiple concentrations of 200, 500 and 1000 cells per plate per irradiation dose. The cells were incubated at 37 °C and 5% CO_2_ for 10–12 days, after which they were stained with clonogenic assay stain solution (50% methanol, 10% acetic acid, 1% *w/v* Coomassie brilliant blue). Colonies (≥50 cells) were counted and the surviving fraction was calculated.

### 4.9. Statistical Analysis

All experiments were at least performed three times except for the qPCR array experiment screening for CSC related genes which was performed in duplicate for each sample and each cell line. However, the gene of interest was validated separately by qPCR of at least three experiments. Shown data are presented as mean and shown error bars represent standard deviation unless otherwise stated. Groups were compared with Student’s *t*-test. IHC results of PTCH1 were compared with the Fishers exact test and of SHH were compared with the chi-square test for trend using SPSS (version 22). A *p*-value <0.05 was considered significant.

## 5. Conclusions

In this study, the potential of targeting the Hedgehog pathway to enhance the efficacy of esophageal cancer was investigated. Enhanced levels of Sonic Hedgehog and its receptor Patched1 were observed in microresidual disease biopsies compared to biopsies from control patients. In vitro modulation of the HH pathway efficiently altered CSC populations, while inhibition of the pathway can be used to enhance sensitivity to common esophageal cancer treatments. The HH pathway therefore offers a potentially attractive target to enhance esophageal cancer treatment.

## Supplementary Materials

The supplementary materials are available online at https://www.mdpi.com/2072-6694/11/6/821/s1. Table S1: Fold change of expression in important cancer stemness-related genes. Figure S1: Gating strategies of the FACS experiments. (A) The left 3–15% proportion of CD44+/CD24- cells and the right 3–15% proportion of CD44+/CD24+ cells were sorted for the induction of the HH pathway by SHH. (B) CD44 and CD24 expression in OE21 and OE33 cells of vismodegib (5nM) sample compared to control sample. Gates of all samples were set individually after correcting for aspecific binding by running isotype controls. Figure S2: Viability curves of OE21 and OE33 after treatment Vismodegib Relative number of viable cells after different concentrations of vismodegib treatment compared to no vismodegib treatment (control). Viable cells were counted with trypan blue. 5 nM was subsequently chosen as the concentration to use.
