# Supplementary material for: Hedgehog Pathway as a Potential Intervention Target in Esophageal Cancer
Source: Cancers (Basel). 2019 Jun 13;11(6):821. doi: 10.3390/cancers11060821 (PMC6627376; doi:10.3390/cancers11060821)
Supplement: Supplementary file 1 [file cancers-11-00821-s001.pdf]

Gene	OE21 CD44^{+}/CD24^{-}	OE21 CD44^{+}/CD24^{+}	OE21 Control
ITGA4	13.13	8.32	1
PTCH1	9.31	3.67	1
NOTCH1	6.49	5.14	1
ID1	5.16	4.26	1
ABCG2	4.58	1.45	1
MYC	3.66	2.36	1
ALDH1A1	3.16	1.01	1
EPCAM	2.59	1.39	1
TAZ	2.56	1.45	1
EFTA	2.44	1.34	1
WWC1	2.41	1.38	1
FGFR2	2.12	1.26	1
**Gene**	**OE33 CD44^{+}/CD24^{-}**	**OE33 CD44^{+}/CD24^{+}**	**OE33 Control**
ID1	4.79	3.96	1
PLAUR	2.54	1.98	1
PTCH1	2.37	1.71	1
ITGA6	2.14	1.4	1

Supplementary Table 1: Fold change of expression in important cancer stemness-related genes. A dedicated qPCR array for 84 genes known to be important in cancer stemness was performed and only genes with >2-fold up-regulated in CD44+/CD24- CSC population compared to control in OE21 and OE33 cells are shown. Control refers to OE21 or OE33 cells which were previously harvested from xenograft models, as these are more heterogeneous than cultured OE21 or OE33 cells alone.

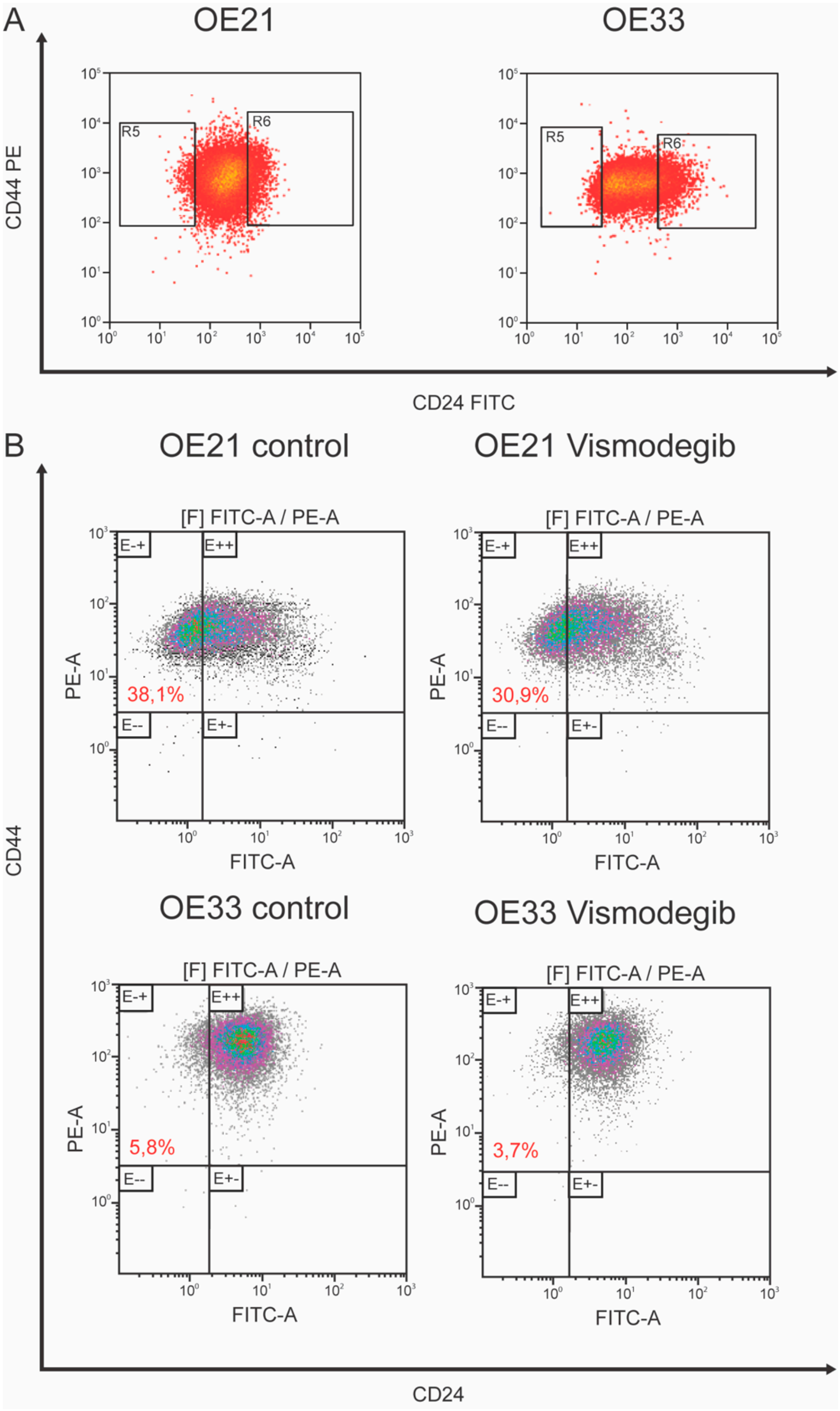

Figure 1. Gating strategies of the FACS experiments. (a) The left 3-15% proportion of CD44+/CD24- cells and the right 3-15% proportion of CD44+/CD24+ cells were sorted for the induction of the HH pathway by SHH. (b) CD44 and CD24 expression in OE21 and OE33 cells of vismodegib (5nM) sample compared to control sample. Gates of all samples were set individually after correcting for aspecific binding by running isotype controls.

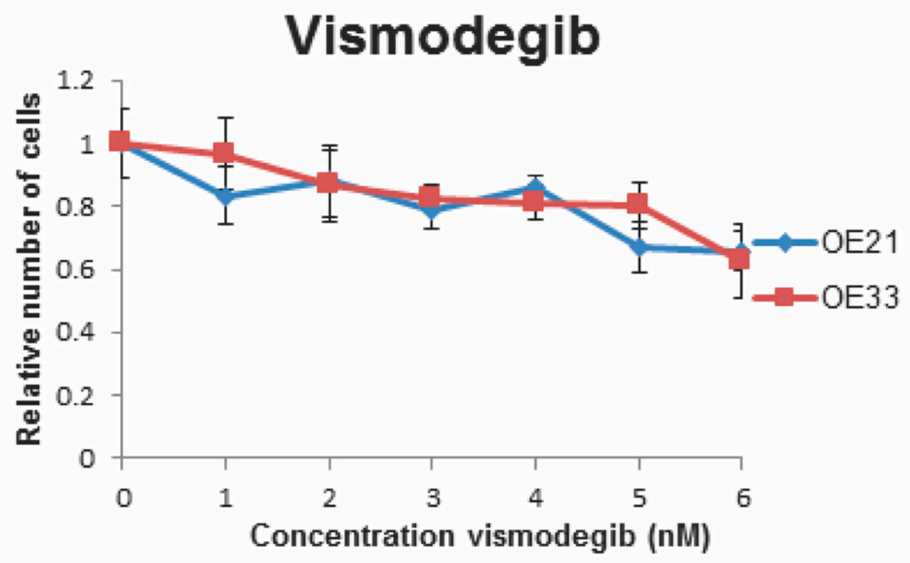

Supplementary figure 2: Viability curves of OE21 and OE33 after treatment Vismodegib
Relative number of viable cells after different concentrations of vismodegib treatment compared to no vismodegib treatment (control). Viable cells were counted with trypan blue. 5nM was subsequently chosen as the concentration to use.
